# Impact of food allergy on health-related quality of life: A case report of 2 patients with adult-onset IgE mediated food allergy

**DOI:** 10.5339/qmj.2023.sqac.8

**Published:** 2023-11-19

**Authors:** Aqel Sami, Asaad Omer Imameldin, Tayseer Ibrahim

**Affiliations:** ^1^Adult Allergy and Immunology Division, Department of Medicine, Hamad Medical Corporation, Doha, Qatar Email: SAqel@hamad.qa

## Abstract

Introduction: Food allergy incidence is increasing, and reactions can be life-threatening. Food allergies significantly impact patients’ and families’ quality of life (QoL).

Here, we describe two cases with adult-onset IgEmediated food allergy impacting their physical and psychological health and affecting their quality of life.

Case Report: *Patient 1*: A 29-year-old lady, previously healthy, presented with a history of sneezing, shortness of breath, and eye and lip swelling shortly after drinking cow milk at the age of 28 years. She had multiple episodes of similar symptoms after consumption of dairy products. A positive blood test confirmed Cow’s Milk allergy (Table 1). She was very anxious and concerned about accidental exposure and even avoided serving milk to her children. She scored 5.2/7 on the Food Allergy Quality of Life Questionnaire-Adult Form (FAQLQ-AF). The patient currently avoids dairy products and carries Adrenaline autoinjectable (AAI), with no reported anaphylaxis.

*Patient 2*: A 46-year-old man with a history of type 2 DM was evaluated for recurrent urticaria. Further history revealed recurrent episodes of urticaria and angioedema for the past few years one to two hours after wheat intake; the most recent episode was typical for anaphylaxis, and he denied any exercise or presence of other co-factors. Allergy testing confirmed wheat allergy (Table 1). He scored 4.9/7 on FAQLQ-AF. He was concerned about having another anaphylaxis. He was instructed to avoid wheat and to carry AAI, after which no further attacks were reported.

Intervention: Identifying the food allergens for both patients alleviates some of their anxiety. Moreover, having an AAI on hand and training on how and when to use it led to increased safety feeling, although initially, they were hesitant to carry AAI. Continuous assessment will be carried out during clinic visits to ensure improved QoL.

Conclusion: Several factors affect the extent of food allergy’s impact on patients’ QoL, like age, type, and number of food allergens.

Physicians must be aware of the psychological burdens faced by adults with food allergies and implement strategies to address and improve patients’ QoL through counseling, proper education, and psychotherapy if needed.

## Figures and Tables

**Table 1.** Demographic and Clinical Characteristics of two patients with adult-onset Food allergy.

**Demographic and clinical characteristics**	**Patient 1**	**Patient 2**
Age at diagnosis (years)	29	46
Age of symptom’s onset (years)	28	42
Previous history of atopy or allergies	No	No
History of anaphylaxis	Yes	Yes
Total IgE (kunits/L)	358	797
Specific IgE for Food allergens (kunits/L)	Cow’s milk: 49.20	Wheat: 2.76
	Casein milk: 2.83	Gluten: 4.89
	Cheddar cheese: 4.81	
	mold cheese: 26.50	
Skin prick test for food allergens	NA	Wheat: 6 mm (Histamine:10 mm, Negative control:0 mm)
Diagnosis	IgE-mediated Food allergy to cow’s milk	IgE-mediated Food allergy to wheat

*NA: Not available*

